# Population Genetics of the Invasive Red Fox, *Vulpes vulpes,* in South-Eastern Australia

**DOI:** 10.3390/genes12050786

**Published:** 2021-05-20

**Authors:** Kalynda M.-A. Watson, Katarina M. Mikac, Sibylle G. Schwab

**Affiliations:** 1 School of Earth, Atmospheric and Life Sciences, Faculty of Science, Medicine and Health, University of Wollongong, Northfields Ave, Wollongong 2522, Australia; kw899@uowmail.edu.au; 2 School of Chemistry and Molecular Biosciences, Faculty of Science, Medicine and Health, University of Wollongong, Northfields Ave, Wollongong 2522, Australia; schwab@uow.edu.au; 3 Illawarra Health and Medical Research Institute, Northfields Ave, Wollongong 2522, Australia

**Keywords:** red fox, single nucleotide polymorphisms, population genetics, fox control

## Abstract

The use of genetic information in conservation biology has become more widespread with genetic information more readily available for non-model organisms. It has also been recognized that genetic information from invasive species can inform their management and control. The red fox poses a significant threat to Australian native fauna and the agricultural industry. Despite this, there are few recently published studies investigating the population genetics of foxes in Australia. This study investigated the population genetics of 94 foxes across the Illawarra and Shoalhaven regions of New South Wales, Australia. Diversity Array sequencing technology was used to genotype a large number of single nucleotide polymorphisms (*N* = 33,375). Moderate genetic diversity and relatedness were observed across the foxes sampled. Low to moderate levels of inbreeding, high-levels of identity-by-state values, as well as high identity-by-descent values were also found. There was limited evidence for population genetic structure among the foxes across the landscape sampled, supporting the presence of a single population across the study area. This indicates that there may be no barriers hindering fox dispersal across the landscape.

## 1. Introduction

The European red fox, *Vulpes vulpes,* has been recognised as an invasive species and key threat to Australian fauna, following the deliberate introduction and establishment of the species in southern Victoria about 140 years ago [1]. The ability of the red fox to colonise biomes ranging from deserts to tundra demonstrates local adaptation to a variety of environments and probably aids in the species’ success as an invasive species [2]. Foxes occupy most of continental Australia, except for the northern arid and tropical regions [3]. It is well known that foxes severely impact upon the distribution and abundance of Australian native fauna [4,5]. Their main prey are livestock and native fauna in the critical weight range [6,7,8]. Reintroduction programs of some threatened native mammalian species in this critical weight range (35 g to 5.5 kg) have shown that absence or tight control of foxes can be detrimental to the success of these programs [9,10,11,12]. Foxes compete with native species for habitat and resources and therefore threaten the presence of already declining native populations. Such has been observed in the spotted-tail quoll (*Dasyurus maculatus)*, with a conservation status of near-threatened, where there is extensive overlap in habitat and resource use [13]. Field surveys have confirmed that spotted-tail quoll population densities are greatest in the absence of foxes or where fox sightings are rare [14]. There is no doubt that foxes pose a serious threat to native populations by competition for resources and habitat overlap, and controlling fox populations will be important to the success of any conservation efforts for threatened Australian fauna.

The most widely used method to control foxes remains sodium fluoroacetate (compound 1080) baiting [15]. However, baiting so far has only resulted in temporary reductions of fox populations. As such, further attempts for reducing the number of foxes, informed by increased knowledge on fox biology and genetics, is needed. Drawing on the availability of rapid genomic sequencing technology, it has been suggested that data on genetic diversity and structuring of populations can provide insight into the efficacy of control strategies [16,17,18]. Specifically, population genetics can inform invasive species management efforts through investigation of the connectivity within and between populations [18]; genetic kinship analysis, where individuals can be linked to a kin group rather than a specific population [19]; estimates of genetic diversity [20]; and distinguishing between surviving and reinvading individuals during or after eradication control programs [17].

Thus far, markers used for tracking genetic diversity of wildlife had focused on the use of highly informative microsatellite markers. Based on known dog breeds’ microsatellite loci, Atterby et al. [20] used 14 microsatellite markers in a fox study, aiming at identifying the recent historical movements of foxes in Britain. Zecchin et al. [21] also used 21 microsatellite markers (dog homologues) to investigate the structuring and movement patterns in foxes in southern Europe. Apart from the use of microsatellite markers that are dog homologues, no other studies have used nuclear markers to investigate the population genetics of foxes. There is a need to explore the use of other nuclear markers, such as single nucleotide polymorphisms (SNPs). More recently, SNPs, which had been used in human genetics for many years, and which are easy and relatively cost effective to genotype on a larger scale, have opened up unprecedented opportunities to investigate the genetic diversity and population dynamics of non-model species and wildlife [22]. SNP markers have already been used to investigate invasive species, such as the brown rat [23], feral pigs [24], and raccoon dogs [25], and as such can act as benchmark studies for use of SNPs to study invasive foxes in Australia. SNPs offer a high-throughput method of genotyping DNA, low genotyping error rates, high frequency of occurrence throughout the genome, and high accuracy of parallel detection [22,26]. To date, no studies in Australia have used SNPs to investigate the population genetics of the fox. Since the red fox genome has recently been sequenced [27], whole-genome analysis of the fox has become feasible, offering an opportunity to investigate the population genetics of the fox in Australia in more detail. This could potentially help to better understand the connectivity and population dynamics of red foxes in Australia, with the prospective of using this information to inform future fox control strategies.

Here, we report on the genetic diversity and genetic relatedness of red foxes across an agricultural landscape in south-eastern Australia, using SNPs. The aim of this study was to improve the understanding of red fox genetics in south-eastern Australia. Investigating the population genetics of this key invasive species is a fundamental step in improving fox control, and therefore reducing their negative impact in Australia.

## 2. Methods

### 2.1. Study Location and Tissue Collection

**Figure 1:**
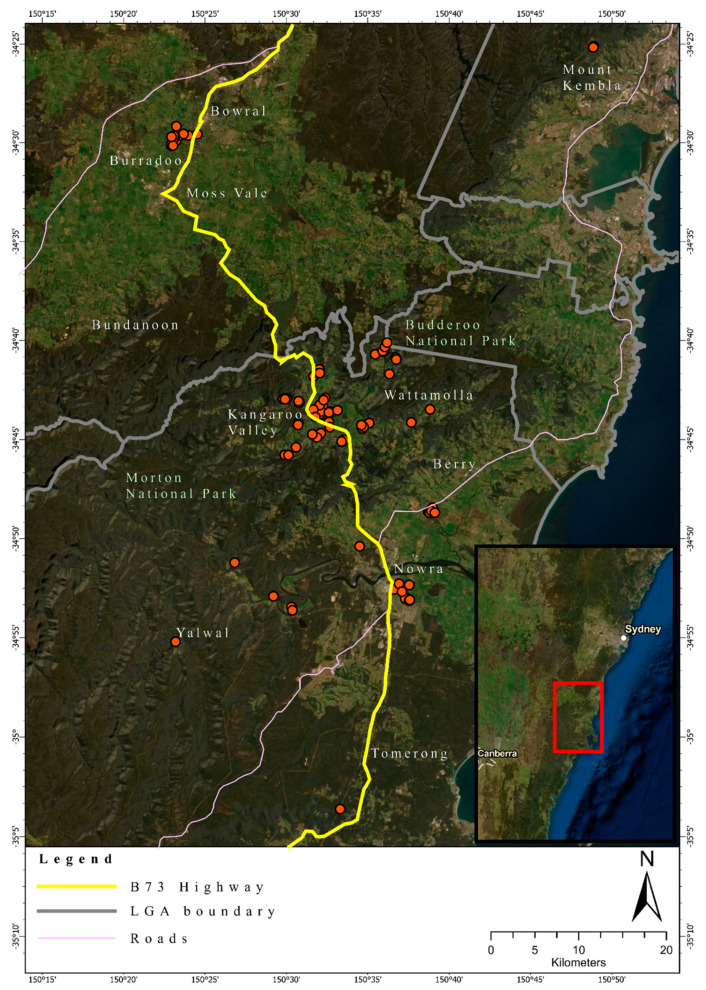
Geographic location of the foxes sampled in this study in south-eastern Australia. Round red dots indicate the location of an individual fox. A total of 94 foxes (Female = 29; Male = 51; Unknown = 14) were sampled across a one-year period. The map was generated using ArcGIS Pro v2.4.

Fox ear tissue of 94 foxes, collected over the timeframe of one year, was donated by pest controllers contracted by the Berry to Budgong Fox Control Program [28]. Tissue samples were stored in 96% ethanol at −20 °C prior to DNA extraction and pending further genetic analyses. Basic demographic (weight, sex, approximate age, date of collection, and health condition) data were provided for 80 foxes, and location (Global Positioning System (GPS) coordinates) data were provided for all foxes sampled (Table S1). The location of the foxes sampled spanned a large geographic area of the Illawarra and Shoalhaven regions of south-eastern NSW, Australia, ranging approximately 75 km from north to south and covering an area of 1170 km^2^ (Figure 1).

### 2.2. DNA Extractions

Approximately 0.5 cm^2^ ear tissue was used to extract genomic DNA, using a commercially available Qiagen DNeasy DNA extraction kit (QIAGEN, Hilden, Germany), following the recommended manufacturer’s protocol. Extracted DNA was subjected to quality control measures, including visualization by agarose gel electrophoresis and spectrophotometric quantification at 260 nm using a Nanodrop^TM^ 2000 spectrophotometer (Thermo Fisher Scientific, Riverstone, NSW, Australia).

### 2.3. Genotyping

Genotyping of the 94 foxes was undertaken by Diversity Arrays Technology Canberra (DArT) Pty Ltd. (Canberra, Australia) using DArTseq technology. The method uses complexity reduction through the use of restriction enzymes, as well as preferentially targeting low-copy genomic regions over repetitive DNA fragments, increasing assay sensitivity, and allowing for detection of a high number of informative SNPs across the genome [29,30]. The red fox genome assembly VulVul2.2, as available from NCBI [31], was used as a comparison sequence for SNP annotation. A quality control protocol, as described below for the SNP markers identified by DArT, was performed before the consolidated list of SNPs was used for subsequent population genetic analysis.

### 2.4. Quality Control and Data Analysis

The DArT SNP data and associated basic data provided were converted into a genlight object using the package adegenet [32] to assist processing with the package dartR v1.1.11 [33]. SNPs were filtered to include only those with a >90% call rate, SNPs with a Hardy–Weinberg Equilibrium without Bonferroni correction with significance of *p* < 0.05, and those with >1% minor allele frequencies, using the package dartR in R v4.0.0 [34].

The filtered dataset was used to estimate observed (*H_O_*) heterozygosity and expected (*H_E_*) heterozygosity using dartR and HIERFSTAT v0.04-22 [35]. Nei’s pairwise genetic differentiation (*F_ST_*) [36] was calculated using HIERFSTAT. For the filtered dataset, minor allele frequencies and the proportion of missing SNPs per genotype were calculated using PLINK v1.07 [37]. The inbreeding coefficient (*F_IS_*) was calculated using PLINK after having randomly removed one individual from potential first-degree relative pairs. The program STRUCTURE v2.3.4 [38] was used to explore the most likely population substructure and model. The admixture ancestry model with correlated allele frequencies was used, assuming a uniform prior and without prior population information. The length of the burn-in period was set to 10,000 iterations. The number of Markov Chain Monte Carlo repetitions was set to 1,000,000. The optimum number of clusters was determined by performing runs at *K* = 1 to 9 for 10 iterations. Structure Harvester v0.6.94 [39] was used to calculate Δ*K* to explore the number of possible genetically distinct clusters. In addition, the Principal Coordinate Analysis (PCoA), as available in the dartR program package, was used to allocate single foxes into potential genetic clusters. Finally, PLINK v1.07 was used to explore any study-wide significant allele frequencies (*p* < 0.0001) between the two postulated genetic clusters.

Sex-biased dispersal was tested for using the R package HIERFSTAT. The test was run using the mean allelic index count model based on the method presented by Goudet et al. [40] for 10,000 iterations. The test was performed for both the one-population model and a two-genetic-cluster model, henceforth referred to as the two-cluster model. Significance was considered at a nominal value of *p* < 0.05.

Pairwise identity-by-state (IBS) was calculated using PLINK. Identity-by-descent values were calculated using PLINK to determine the potential kinship within the sampled foxes. The type of relationship was estimated in consultation with the demographic data available for the foxes (see Section 2.1). This included examining the approximate age range of the foxes, whether they were collected on the same day in the same location, and consultation with fox reproduction biology to determine if foxes were sexually mature at that age, i.e., greater than one year old [41]. A Mendelian error test was performed to determine whether the parent–offspring relationships predicted were a likely event, tolerating a Mendelian error rate of 2% [37,42].

## 3. Results

### 3.1. Quality Control

The DNA of 93 foxes was successfully genotyped. One sample did not return genotypes and was therefore excluded from further analysis. In total, 33,735 SNPs were reported. Filtering for a 90% call rate removed 7092 SNPs; filtering for Hardy–Weinberg Equilibrium at a nominal α value of α = 0.05 (without Bonferroni correction for multiple testing) removed a further 8202 SNPs. A final minor allele frequency filter for SNPs with frequencies <1% excluded a further 543 SNPs. Minor allele frequencies for the remaining 17,898 SNPs ranged from 0.012 to 0.5. The large majority (49.7%) of SNPs had minor allele frequencies >0.15 and <0.4, indicative of having a high information content for genetic analysis. Complete genotyping was observed for 11,307 or 63% of the SNPs. Only four foxes had more than 5% (but less than 8%) of their genotype missing.

### 3.2. Heterozygosity of the One- and Two-Cluster Models

**Figure 2:**
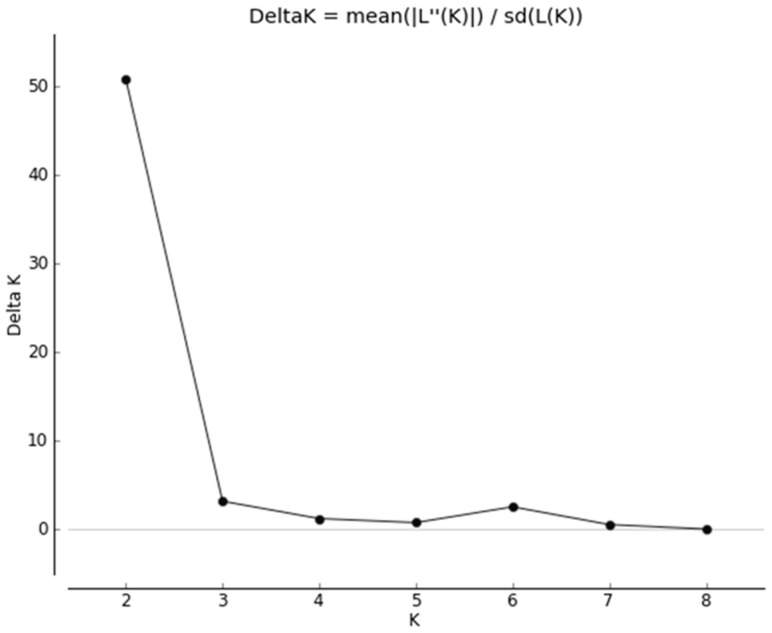
Results from Structure Harvester analysis to reveal the most likely value of *K* based on the STRUCTURE results.

The ΔK plot (Figure 2) indicated a sharp peak at *K* = 2.

Taking into account that Structure Harvester cannot accurately estimate the number of genetic clusters if *K* = 1, we decided to model two different scenarios. The first was a model for a single genetic cluster in which all foxes (*N* = 93) were grouped. Assuming this model, a moderate heterozygosity (*H_O_*) was observed (*H_O_* = 0.280). Moderate genetic diversity, estimated by expected heterozygosity (*H_E_*), was observed across all foxes (*H_E_* = 0.302). Values of inbreeding were estimated (*F_IS_* = 0.057 (−0.277–0.343)), with some larger values of inbreeding observed for a small portion of foxes (*F_IS_* > 0.2, *N* = 6).

**Figure 3:**
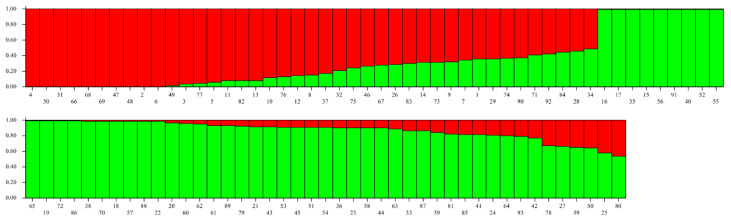
STRUCTURE bar plot for *K* = 2 using a model based on admixture with correlated allele frequencies. Fox identities are represented by vertical bars. Distinct colours of the bars (red and green) represent the proportion of admixture (Q), or ancestry. Fox ID’s are present on the *X*-axis and are ordered according to the Q values presented on the *Y*-axis. N.B., the *X*-axis is continuous.

The second scenario comprised two genetic clusters of foxes differentiated by allele frequencies as determined by STRUCTURE and PCoA (Figure S1). STRUCTURE revealed a *K* = 2 best-fit genetic cluster model, both from the log probability (LnP) and highest Δ*K* (Δ*K* = 50.8) (Figure 3).

**Figure 4:**
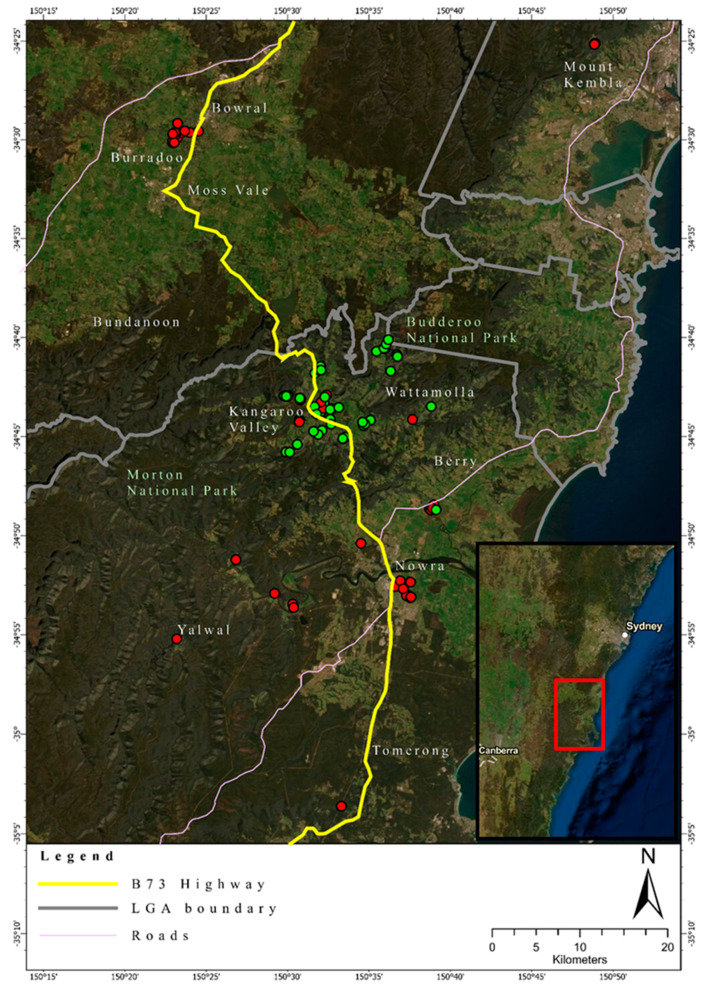
Geographic location of the foxes sampled in this study in south-eastern Australia. Red dots indicate the location of foxes allocated to *Genetic* *Cluster 1*. Green dots indicate location of foxes allocated to *Cluster 2*. The map was generated using ArcGIS Pro v2.4.

Foxes were subsequently assigned to these two hypothetical genetic clusters based on differences in allele frequencies. The number of foxes assigned to *Cluster 1* was 42 (24 males, 4 females, 14 unknown sex) and to *Cluster 2* was 51 individuals (28 males, 23 females), respectively. Foxes belonging to *Cluster 2* were mainly sourced from the area of the Kangaroo Valley, whereas foxes from *Cluster 1* were more identified either north or south of the Kangaroo Valley (Figure 4).

Estimated *H_O_* values varied minimally, with estimated values found to be 0.297 for *Cluster 1* and 0.290 for *Cluster 2*. Similarly, *H_E_* values varied minimally, with estimated values found to be 0.311 for *Cluster 1* and 0.309 for *Cluster 2*. There was some indication of inbreeding, as measured by *F_IS_,* with a larger value range obtained for *Cluster 1*. This value was averaged to be 0.041, with a range of −0.283 to 0.300 for *Cluster 1* (Table 1). *Cluster 2* had an average *F_IS_* value of 0.057, with a range of −0.018 to 0.193. Genetic differentiation measured by *F_ST_* was estimated to be at a value of 0.018 (Table 1).

In an attempt to support the two-cluster model, we compared the allele frequencies for all SNPs in both hypothetical clusters using χ^2^ tests. We found 19 of the 17,898 SNPs revealing study-wide statistically significant differences (*p* < 2.8 × 10^−6^) in allele frequencies (Table S2).

### 3.3. Relatedness

The pairwise relatedness values in the IBS matrix, which compares the distance between every two possible sample combinations, ranged from 0.709 to the highest value of 0.874. The highest value obtained from the IBS relatedness matrix was obtained for two single foxes, both sourced from *Genetic Cluster 1*, south of Kangaroo Valley (IBS relatedness = 0.874) (Figure 4). The overall mean IBS relatedness value present within the fox samples was approximately 0.740, indicating additional evidence for high genetic relatedness across the foxes sampled.

We used PLINK to calculate the IBD and IBS values to construct potential lineages within the foxes. The following key parameters were observed: A Z0 value referring to the proportion of no alleles shared between two individuals. A Z1 value referring to the proportion for which one allele is shared between the two individuals. Finally, a Z2 value referring to the proportion for which two (both) alleles are shared between the two individuals. A $\hat{\pi }$ value (which is a weighted value for Z1 and Z2 and as such reflects an estimate for the proportion of IBD) of approximately 0.5 indicates a potential first-degree relative. This was then deemed to be a potential sibling or parent–offspring relationship. Applying an IBD-$\hat{\pi }$-value of >0.45 and an IBS value of >0.8, we were able to identify 13 potential close (possibly first-degree) kinship relations, comprising of a total of 22 foxes. Together with basic information on age, sex, location of fox collection, and information from STRUCTURE, using the two-cluster model, we explored the nature of these kinships further. We were able to speculate on three small family kinships with a probable first-degree relationship between the foxes (Table 2).

These family kinships were found to be sampled less than 1.6 km of their respective kinship groups. All of these assumed pedigrees were dyads, i.e., one parent and one or two offspring. We tested these kinships by constructing ‘family trees’ and tested the Mendel error rates (Table 2). Compared to randomly constructed family dyads, the Mendel error rates for the established pedigrees were around 5 to 10 times less, providing some support for these established pedigree structures.

### 3.4. Sex-Biased Dispersal

Sex was not provided for 14 foxes; therefore, these foxes were excluded from this analysis. The HIERFSTAT test was performed assuming a one-cluster model and a two-cluster model, independently. Significance was considered at a nominal value of *p* < 0.05. Sex-biased dispersal was found to be not significant, neither for the one-cluster model (*p* = 0.838) nor the two-cluster model (*p* = 0.337).

## 4. Discussion

The aim of our study was to explore the population genetics of the red fox in a confined region (1170 km^2^ landscape) of south-eastern Australia using SNPs. We were able to collect DNA from the ear tissue of 94 red foxes, which were donated to us by hunters from a regional fox control program. We employed a rigorous genotyping data quality control process before analysing the data, making sure that we did not introduce any unintentional bias. We demonstrated that the foxes sampled most likely belonged to one panmictic population, with moderate evidence for reduced genetic diversity and moderate evidence for genetic relatedness within this sample.

Genetic diversity was estimated using HIERFSTAT. Our data indicated some limited genetic diversity of the studied fox population. A more recent study by Walton et al. [43], using fox samples in Sweden and which had been using a limited number of SNPs, reported an average estimated heterozygosity of 0.450. This value is higher than our observed value for *H_E_* of 0.302; indeed, the value reported by Walton et al. [43] is closer to those values reported for microsatellite *H_E_*, which range from approximately 0.46 to 0.75 [20,44]. Therefore, estimated values of heterozygosity observed in this study may represent a loss of genetic diversity when compared to fox populations in the Northern Hemisphere. Similarly, moderate values of the inbreeding coefficient *F_IS_* = −0.283 to 0.300 were estimated here. This indicates that the sampled foxes investigated may have a reduced gene pool due to a moderate degree of genetic relatedness. This was also found in the IBS analysis, where foxes had high values of pairwise IBS distances. The reduced heterozygosity of the sampled foxes may also indicate the effects of range expansions of the founder individuals first introduced into Australia [44,45]. It is thought that eight successful releases of foxes in Australia in the 1860–1870s established, which would become one of Australia’s most pervasive and threatening invasive species [1]. The net result is that the introduction of foxes into Australia would have reduced genetic variation and increased genetic drift effects as the founders, and the surviving foxes following establishment, would carry a smaller proportion of the total genetic variation of the source population from the UK (bottleneck). To determine whether the reduced gene pool is due to founder populations of foxes or due to the success of current fox control programs, future studies should also co-analyse DNA from historical samples of foxes in Australia. Historical samples may include museum specimens, or DNA from foxes in the UK, which are ancestral populations of those in Australia [46]. Additionally, the reduced heterozygosity for this sample of foxes may indicate the effects of current fox control efforts. Through decreasing the gene pool available for foxes to breed freely, foxes may have become more related and therefore have lower genetic diversity. Reduced genetic diversity due to founder events is commonly reported for other introduced species globally. This has been observed in brush-tail possums (*Trichosurus vulpecula)* in New Zealand [47], sugar gliders (*Petaurus breviceps)* in Tasmania [48], and dromedary camels (*Camelus dromedarius)* in central Australia [49].

When exploring various population models using STRUCTURE, our study did not find strong evidence for a two-cluster model. Specifically, the *F_ST_* estimated values and a lack of a difference in heterozygosity values supported one panmictic population across the 1170 km^2^ area used in this study. This interpretation was supported by a limited number of loci, which revealed study-wide significant differences in minor allele frequencies between an experimentally constructed two-cluster model. Only 19 of the 17,898 loci used in this study showed significant differences in minor allele frequencies between the two postulated genetic clusters, as determined by association analysis in PLINK. We would like to point out that all SNPs in our study were subjected to strict quality control measures. Specifically, we used stringent inclusion criteria for genotype distribution expectations according to Hardy–Weinberg equilibrium. While the number of male and female foxes is significantly different for the two hypothetically constructed genetic clusters (*Cluster 1*: 24 males and 4 females; *Cluster 2*: 28 males and 23 females; Yates-corrected χ^2^ = 6.320, *p* = 0.012), the genotype distribution for these 19 loci does not seem to be related to sex chromosomal location. Therefore, our study is in alignment with what is expected, as the observed genetic clusters are in close geographic proximity. Given the very low number of these SNPs with such dramatic differences suggests a lack of landscape barriers that otherwise could limit gene flow and dispersal across the region investigated, enabling unmitigated gene flow across the landscape.

The study site is geographically and ecologically characterized by a relatively narrow coastal plain and an escarpment, which rises in elevation up to about 450 m above sea level, opening towards the Southern Highlands. The escarpment, with pronounced valleys, provide eastward drainage for rivers or creeks draining through wet/dry sclerophyll vegetation types. The coastal plain and the highlands are mostly cleared agricultural land with highly fragmented remnant native vegetation. While it may be possible that the escarpment acts as a landscape-scale barrier to the foxes east–west dispersal, it should be noted that this is mitigated by the numerous roads and highways within this location. For example, when main roads are taken into consideration, it is important to note the clustering of foxes near a single location in the Kangaroo Valley. The Kangaroo Valley cluster comprised mainly samples from *Cluster 2* with a limited number of samples from *Cluster 1*, whereas the samples collected close to the town of Nowra, and the samples collected close to the town of Bowral, comprised exclusively of foxes that were allocated to *Cluster 1*. Therefore, foxes may be using the main road that connects these towns as a movement corridor. This information is consistent with findings from previous studies that show fox preferences for manmade roads and tracks [50,51]. However, it is important to also consider that this road was used by the pest contractors when controlling foxes and it may be the movement pattern of the pest controllers that has been revealed and not that of the foxes per se. Future studies should be conducted to confirm the use of movement patterns of red foxes within this area using remote cameras to confirm these movement patterns. This would benefit knowledge about how foxes are using the landscape to travel within the region, and simultaneously allow for the targeting of known fox movement corridors for control [52].

When exploring the possible sex-biased dispersal of foxes, no supportive evidence was found. Although it is stated otherwise, in that male foxes will disperse greater distances from their birth den, the results of this study are not consistent with the published literature [53,54]. Given that our study area and the number of foxes were quite small, we may not have had enough power to draw valid conclusions regarding sex-biased dispersal solely supported by genetic data alone.

## 5. Management Implications and Conclusions

We have shown that across a landscape-scale study in south-eastern Australia, there are insufficient geographic barriers to mitigate geneflow of the foxes sampled. Long-term fox control should focus on identifying key local geographic source populations and concentrate control efforts in these locations. A push toward reduction of gene flow, the structuring of populations across the landscape, and allowing genetic drift to assist in control may assist in fox control [55]. Similarly, implementing genetic monitoring of the invasive species should be a preliminary step in the management process to assess the effectiveness of control efforts [17,56]. Through ongoing observations of reduction in genetic diversity and increases in genetic relatedness, this may indicate the success of control strategies [55]. Similarly, the implementation of genetic monitoring programs can investigate source populations if reinvasion into an area occurs [17], and therefore if gene flow is occurring from source fox populations. As such, genetic monitoring can inform where in the landscape control should be targeted to reduce gene flow.

The goal of control in the region should be to reduce gene flow across the region and to strive for the genetic isolation of foxes. The use of natural genetic control to promote genetic drift and allowing selective pressures through the reduction of advantageous alleles may allow for the reduction of fox population densities and a shift to genetically structured populations.

## Figures and Tables

**Table 1:** Summary table of the statistics associated with fox cluster assignment. *N* refers to the number of foxes allocated to the respective cluster; *H_O_* refers to the total mean of the estimated observed heterozygosity per cluster; *H_E_* refers to the total mean of the estimated expected heterozygosity per cluster; *F_IS_* is the estimated inbreeding coefficient per cluster; *F_ST_* is the estimated pairwise *F* statistic estimated for the two clusters.

Genetic Cluster	*N*	*H*_O_	*H*_E_	*F_IS_*	*F_ST_*
*Cluster 1*	42	0.297	0.311	0.041 (−0.283 to 0.300)	0.018
*Cluster 2*	51	0.290	0.309	0.057 (−0.018 to 0.193)	

**Table 2:** Kinship constructs according to information from IDS, IBD ($\hat{\pi }$), STRUCTURE, and basic biology and location data.

Pedigree	FoxID (Estimated Age)	IBS	$\hat{\pi }$	Cluster Origin According to Structure	Type of Relationship	No. of Mendel Errors (%)
1	16 (<1 year) 17 (<1 year)	0.86	0.54	2	offspring of same mating event	N/A
2	30 (2–3 years) 31 (<1 year)	0.86	0.52	1	parent–offspring relation (dyad)	147 (0.82)
3a	66 (>5 years) 68 (3–4 years)	0.84	0.47	1	parent–offspring relation (dyad)	147 (0.82)
3b	66 (>5 years) 69 (4–5 years)	0.84	0.44	1	parent–offspring relation (dyad)	139 (0.77)
3c	68 (3–4 years) 69 (4–5 years)	0.87	0.53	1	offspring of potentially two mating events in two breeding seasons	N/A

## Data Availability

The data presented in this study are available on request from the corresponding author.

## Supplementary Materials

The following are available online at https://www.mdpi.com/article/10.3390/genes12050786/s1. Figure S1. Principle Co-ordinate Analysis plot of the genetic distances between two populations, *Genetic Cluster 1* and *Genetic Cluster 2*. The PCoA between the first and second axes covers 5.8% of the variation in the data. Red points indicate membership to *Genetic Cluster 1* (*N* = 42). Green points indicate membership to *Genetic Cluster 2* (*N* = 51). Table S1. Basic data that was provided with the shot foxes, including the coordinates where they were shot, approximate age, sex, weight, and condition of health. Table S2. Results from association analysis on PLINK. SNP ID refers to the SNP identifier supplied from DArTseq. Polymorphism refers to the alternate and SNP genotypes. Minor allele refers to the allele with the lower allele frequency in the total population. Allele frequency–*Cluster 1* refers to the allele frequency for the genotypes of foxes in C*luster 1*. *Allele frequency–Cluster 2* refers to the allele frequency for the genotypes of foxes in *Cluster 2*. *p-value* refers to the significant *p*-value of the test with Bonferroni correction (*p* < 0.0001). *** = *p* < 0.0001
