# Supplementary material for: Population Genetics of the Invasive Red Fox, Vulpes vulpes, in South-Eastern Australia
Source: Genes (Basel). 2021 May 20;12(5):786. doi: 10.3390/genes12050786 (PMC8161170; doi:10.3390/genes12050786)
Supplement: Supplementary file 1 [file genes-12-00786-s001.zip › genes-1168309-supplementary.pdf]

genes

Supplementary Material:

Figure S1:

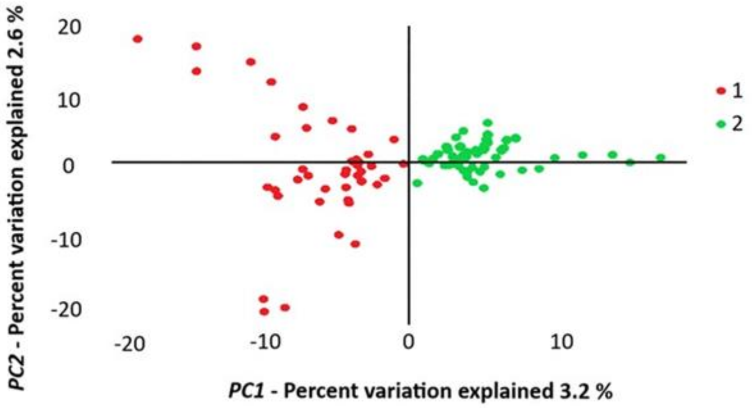

Figure S1. Principle Co-ordinate Analysis plot of genetic distances between two populations, *genetic cluster 1* and *genetic cluster 2*. PCoA between first and second axes covers 5.8 % of the variation in the data. Red points indicate membership to *genetic cluster 1 (N*=42*)*. Green points indicate membership to *genetic cluster 2 (N*=51*)*.

Table S1: Basic data that was provided with shot foxes, including coordinates where shot, approximate age, sex, weight, and condition of health.

Variable	Description
**Individual**	**The number assigned to the individual for further analysis based on the chronological order that the fox was shot by the hunter.**
Date	The date in DD/MM/YY format that the fox was shot.
Latitude	Latitudinal coordinates that the fox was shot in degrees.
Longitude	Longitudinal coordinates that the fox was shot in degrees.
Age (years)	The approximate age in years of the shot fox as estimated by the licensed hunter—opinion of the hunter based on age biology of the fox, distinguishing features based on age. Not provided for 14 foxes.
Sex	The sex of the shot fox—Male or Female. Not provided for 14 foxes.
Weight (kg)	The approximate weight of the shot fox in kilograms (kg). Not provided for 14 foxes.
Condition	The visual condition of health that the shot fox was found in—opinion of the hunter based off presence of mange, excessive scarring due to fighting, size of the fox. Not provided for 14 foxes.

Individual	Date	Latitude	Longitude	Weight (kg)	Age range (years)	Sex	Condition
1	13/3/19	-34.4861	150.3874	-	-	-	-
2	13/3/19	-34.49425	150.3905	-	-	-	-
3	13/3/19	-34.49805	150.3873	-	-	-	-
4	13/3/19	-34.49399	150.39164	-	-	-	-
5	13/3/19	-34.49386	150.39983	-	-	-	-
6	1/5/19	-34.5026	150.3823	-	-	-	-
7	10/4/19	-34.4948	150.3835	-	-	-	-
8	10/4/19	-34.4926	150.3948	-	-	-	-
9	1/5/19	-34.5016	150.3820	-	-	-	-

Individual	Date	Latitude	Longitude	Weight (kg)	Age range (years)	Sex	Condition
10	1/5/19	-34.4960	150.3853	-	-	-	-
11	1/5/19	-34.5024	150.3840	-	-	-	-
12	1/5/19	-34.4928	150.4090	-	-	-	-
13	1/5/19	-34.4940	150.3848	-	-	-	-
14	1/5/19	-34.4950	150.3825	-	-	-	-
15	13/2/19	-34.715785759	150.500489752	3.2	<1	Female	Good
16	13/2/19	-34.715958893	150.496630691	3.5	<1	Male	Good
17	13/2/19	-34.719232436	150.512963361	3	<1	Female	Poor
18	13/2/19	-34.718488354	150.513607968	5	2 to 3	Male	Excellent
19	13/2/19	-34.756547514	150.510009314	4	<1	Female	-
20	15/2/19	-34.696793598	150.527977524	4.4	2 to 3	Female	Good
21	15/2/19	-34.691024941	150.533802714	2.1	<1	Female	Good
22	15/2/19	-34.694413699	150.532425116	3.6	1 to 2	Male	Excellent
23	15/2/19	-34.694134817	150.534234315	6.1	2 to 3	Male	Excellent
24	19/2/19	-34.735818913	150.512040333	3.6	1 to 2	Female	Good
25	19/2/19	-34.721513995	150.537395499	5.8	2 to 3	Male	Good
26	25/2/19	-34.839770162	150.575043027	6.2	2 to 3	Male	Excellent
27	2/3/19	-34.695051931	150.605503027	5.5	2 to 3	Female	Good
28	8/3/19	-35.060248839	150.555129795	4.5	1 to 2	Male	Good
29	8/3/19	-34.810695990	150.645171468	7.1	4 to 5	Male	Good
30	8/3/19	-34.807431500	150.649778435	6.5	2 to 3	Male	Good
31	8/3/19	-34.811650250	150.646499685	2.5	<1	Male	Good
32	8/3/19	-34.810182120	150.648432387	5	2 to 3	Female	Fair
33	10/3/19	-34.724637610	150.647026137	4.5	1 to 2	Female	Good
34	10/3/19	-34.735562430	150.627900497	5.1	3 to 4	Male	Fair
35	10/3/19	-34.739410854	150.577041464	3.5	1 to 2	Female	Good
**Individual**	**Date**	**Latitude**	**Longitude**	**Weight (kg)**	**Age range (years)**	**Sex**	**Condition**
36	4/5/19	-34.811567249	150.652000072	6.1	3 to 4	Male	Excellent
37	5/5/19	-34.418635680	150.814940020	3.8	1 to 2	Male	Poor
38	7/5/19	-34.746362713	150.530902177	2.6	1 to 2	Female	Poor
39	7/5/19	-34.736185747	150.584867249	7	4 to 5	Male	Excellent
40	7/5/19	-34.740752446	150.577865027	4.5	1 to 2	Male	Good
41	7/5/19	-34.739471218	150.579169247	3.5	1 to 2	Female	Good
42	7/5/19	-34.737881038	150.576725771	6	3 to 4	Male	Good
43	7/5/19	-34.744757205	150.535307583	4.2	1 to 2	Female	Fair
44	7/5/19	-34.748229281	150.531370844	6.8	3 to 4	Male	Excellent
45	16/5/19	-34.745615494	150.526512174	6.3	3 to 4	Male	Excellent
46	25/9/19	-34.853583221	150.447051376	5.1	1 to 2	Male	Good
47	29/10/19	-34.419417243	150.814277788	1	<1	Male	Good
48	29/10/19	-34.419417243	150.814277788	1	<1	Female	Good
49	29/10/19	-34.419417243	150.814277788	0.8	<1	Male	Good
50	2/11/19	-34.715252112	150.539797492	6.7	3 to 4	Male	Good
51	2/11/19	-34.730950245	150.533271811	5.4	2 to 3	Female	Good
52	2/11/19	-34.728835783	150.534361075	6.2	3 to 4	Male	Excellent
53	9/11/19	-34.752005469	150.556783833	3.4	1 to 2	Female	Good
54	9/11/19	-34.750388224	150.558852082	1.2	<1	Male	Good
55	20/11/19	-34.678404434	150.591043014	6.5	4 to 5	Male	Excellent
56	22/11/19	-34.676148024	150.599587367	1.1	<1	Female	Good
57	22/11/19	-34.675798320	150.598768119	6.2	4 to 5	Male	Fair
58	22/11/19	-34.672290065	150.600986345	4.5	1 to 2	Female	Good
59	22/11/19	-34.668572384	150.603101296	2	<1	Male	Excellent
60	22/11/19	-34.681196007	150.611948086	4	1 to 2	Female	Good
61	22/11/19	-34.682811770	150.612512819	0.8	<1	Male	Good
**Individual**	**Date**	**Latitude**	**Longitude**	**Weight (kg)**	**Age range (years)**	**Sex**	**Condition**
62	13/12/19	-34.763070016	150.498376300	1.2	<1	Male	Good
63	13/12/19	-34.763192880	150.502163099	1.3	<1	Male	Good
64	13/12/19	-34.736014354	150.512340234	4.7	1 to 2	Female	Good
65	13/12/19	-34.726470776	150.544913507	1.2	<1	Female	Good
66	8/7/19	-34.883224772	150.623105173	7.8	>5	Male	Excellent
67	8/7/19	-34.883803827	150.621830394	6.3	3 to 4	Male	Good
68	8/7/19	-34.876168479	150.610356705	6.5	3 to 4	Male	Excellent
69	8/7/19	-34.872396263	150.625790686	7	4 to 5	Male	Good
70	27/12/19	-34.727150425	150.544194583	1.5	<1	Male	Good

71	27/12/19	-34.726366450	150.535733790	6.5	3 to 4	Male	Good
72	27/12/19	-34.727162263	150.543440820	4.1	1 to 2	Female	Good
73	27/12/19	-34.720944757	150.534213360	6.5	3 to 4	Male	Excellent
74	21/1/20	-34.871288072	150.615234279	4.6	<1	Female	Good
75	21/1/20	-34.877910890	150.618291845	5	2 to 3	Male	Good
76	21/1/20	-34.885543780	150.625521791	3.9	<1	Male	Good
77	21/1/20	-34.884875482	150.626383414	3.5	<1	Male	Good
78	25/1/20	-34.736302652	150.512331589	4.4	<1	Male	Good
79	25/1/20	-34.724847996	150.527535271	4.6	<1	Male	Good
80	25/1/20	-34.731094556	150.529718681	6.5	2 to 3	Male	Excellent
81	25/1/20	-34.716814158	150.538135391	3	<1	Male	Good
82	28/1/20	-34.890804164	150.505197237	4.8	<1	Male	Good
83	28/1/20	-34.893647339	150.506249091	5	<1	Male	Good
84	30/1/20	-34.881947918	150.486638402	4.8	1 to 2	Male	Good
85	4/2/20	-34.717737648	150.512604190	4.1	1 to 2	Female	Good
86	4/2/20	-34.735404899	150.543589897	4.2	1 to 2	Female	Fair
87	4/2/20	-34.739489553	150.544528245	4.5	2 to 3	Female	Good
**Individual**	**Date**	**Latitude**	**Longitude**	**Weight (kg)**	**Age range (years)**	**Sex**	**Condition**
88	4/2/20	-34.725439711	150.552357881	3.5	<1	Male	Excellent
89	13/2/20	-34.736493050	150.511943674	6	3 to 4	Male	Excellent
90	20/2/20	-34.920093973	150.386539111	3	<1	Female	Good
91	24/2/20	-34.716064113	150.498679995	3.5	<1	Male	Excellent
92	24/2/20	-34.718958770	150.498723788	4	<1	Female	Good
93	25/2/20	-34.737710645	150.511972676	4	<1	Female	Good
94	28/2/20	-34.751638949	150.556488809	5.9	3 to 4	Male	Fair

Table S2. Results from association analysis on PLINK. *SNP ID* refers to the SNP identifier supplied from DArTseq. *Polymorphism* refers to the alternate and SNP genotypes. *Minor allele* refers to the allele with the lower allele frequency in the total population. *Allele frequency – population 1* refers to the allele frequency for the genotypes of foxes in *genetic cluster 1*. *Allele frequency – population 2* refers to the allele frequency for the genotypes of foxes in *genetic cluster 2*. *p-value* refers to the significant p-value of the test with Bonferroni correction ($p < 0.0001$). *** = p <0.0001.

SNP ID	Polymorphism	Minor allele	Allele frequency – *population 1*	Allele frequency – *population 2*	p-value
10725763-16-G/C	G/C	C	0.3611	0	***
10723124-30-T/C	T/C	C	0.6111	0.125	***
100009517-51-T/G	T/G	G	0.7222	0.2024	***
57022061-8-C/T	C/T	C	0.4706	0.06818	***
10712442-5-C/A	C/A	A	0.2778	0	***
100016951-23-C/T	C/T	T	0.4444	0.05814	***
45537018-48-G/A	G/A	A	0.1111	0.6136	***
57022555-47-C/T	C/T	C	0.7222	0.2386	***
100027824-14-C/T	C/T	T	0.3056	0.01136	***
10715172-47-G/T	G/T	G	0.3056	0.01136	***
57020395-40-C/T	C/T	T	0.3611	0.03409	***
100022293-24-G/C	G/C	C	0.3333	0.02326	***
45541004-6-C/T	C/T	T	0.2941	0.01163	***
100035282-6-G/C	G/C	C	0.25	0	***
45544731-33-T/G	T/G	G	0.5	0.1047	***
45544731-33-T/G	T/G	G	0.5	0.1047	***
17675565-12-A/G	A/G	G	0.6944	0.2386	***
45533640-16-G/A	G/A	A	0.7222	0.2614	***
45536391-35-G/A	G/A	A	0.4444	0.07955	***
